# Parental Leave Benefits and Maternal Postpartum Mental Health in Sweden

**DOI:** 10.1001/jamanetworkopen.2025.8062

**Published:** 2025-04-30

**Authors:** Amy Heshmati, Helena Honkaniemi, Sara Fritzell, Sol P. Juárez

**Affiliations:** 1 Centre for Health Equity Studies, Stockholm University–Karolinska Institutet, Stockholm, Sweden; 2 Department of Public Health Sciences, Stockholm University, Stockholm, Sweden; 3 Department of Global Public Health, Karolinska Institutet, Stockholm, Sweden

## Abstract

**Importance:**

Generous parental leave benefits have been associated with better mental health outcomes for parents after childbirth. However, few studies account for preconception mental health and working conditions, the latter being a requirement for generous parental leave benefits.

**Objective:**

To evaluate the association between levels of parental leave benefits and maternal postpartum mental health when preconception mental health, income, and employment status were considered.

**Design, Setting, and Participants:**

This population-based nationwide cohort study included first-time mothers aged 18 to 52 years who gave birth to a live singleton offspring between January 1, 2007, and December 31, 2011. Data were analyzed from March 8 to February 18, 2025.

**Exposure:**

Paid parental leave benefits, including higher level (qualified for earnings-related benefits equal to approximately 80% of their salary) vs basic level (a flat-rate benefit for those not meeting the work requirements for earnings-related benefits).

**Main Outcomes and Measures:**

Nationwide registers were used to perform multivariable logistic regression to calculate the odds of mental health outcomes from different levels of severity (prescription of antidepressants or anxiolytics, specialist outpatient care, and hospitalization) between mothers receiving higher-level and basic benefits. Decomposition analyses using the Karlson-Holm-Breen method were conducted to examine the contribution of preconception mental health, income, and employment status.

**Results:**

The sample included 210 800 first-time mothers (mean [SD] age, 28.6 [5.0] years), of whom 35 255 (16.72%) received basic benefits and 175 545 (83.28%) qualified for higher-level benefits. Mothers receiving basic benefits had higher odds of being prescribed antidepressants or anxiolytics (odds ratio [OR], 1.44; 95% CI, 1.37-1.51), receiving specialist outpatient care (OR, 2.27; 95% CI, 2.13-2.44), and being hospitalized (OR, 2.47; 95% CI, 2.06-2.97) compared with mothers receiving higher-level benefits. Higher odds remained after adjusting for preconception mental health, accounting for a 46.1% decrease for prescribed antidepressants or anxiolytics, 15.1% for outpatient care, and 10.7% for hospitalization. After adjustment for income and employment status, the odds remained elevated for specialist outpatient care (OR, 1.13; 95% CI, 1.02-1.24) and hospitalization (OR, 1.34; 95% CI, 1.03-1.76).

**Conclusions and Relevance:**

In this cohort study of first-time mothers in Sweden, higher-level benefits were associated with better mental health, particularly moderate-to-severe mental disorders. However, since eligibility for higher-level parental leave benefits is contingent on labor market attachment, both improving women’s labor market attachment and relaxing the work requirements for higher-level parental leave could promote women’s postpartum mental health.

## Key Points

**Question:**

Are parental leave benefit levels associated with maternal postpartum mental health when preconception mental health and labor market attachment are considered?

**Findings:**

In this cohort study of 210 800 first-time mothers who gave birth between 2007 and 2011 in Sweden, 17% received basic benefits, while 83% qualified for higher-level benefits, equivalent to 80% of their salary. Mothers receiving basic benefits had significantly higher odds of moderate-to-severe mental health outcomes compared with those receiving higher-level benefits, even after accounting for preconception mental health, income, and employment status.

**Meaning:**

These findings suggest that higher-level parental leave could promote women’s postpartum mental health.

## Teaser

This cohort study evaluates the association between levels of paid parental leave benefits and maternal mental health during the postpartum period among first-time mothers in Sweden while accounting for preconception mental health, income, and employment status.

## Introduction

The transition to parenthood represents a major life event where mothers experience not only biological changes but also challenges related to child rearing, career disruption, and financial pressures due to time off work and lower income.^1^ Parental leave, defined as job-protected leave of absence to take care of a child, may prevent or reduce stress and other mental health symptoms in the postpartum period.

A recent international literature review^2^ concluded that parental leave has protective effects for mother’s mental health in the postpartum period and beyond. However, this review also highlighted the need for observational studies to consider preconception mental health, as prospective evidence shows that a large proportion of postpartum depression stems from preconception mental health.^3,4^ Similarly, few studies examining the association between parental leave benefits and maternal mental health control for employment factors,^5,6^ which is important given that eligibility for these benefits is subject to strict work requirements.

Observational studies evaluating the association between mothers’ parental leave and postpartum mental health have relied on survey data^5,6,7,8,9,10,11,12,13,14,15,16,17,18,19,20,21,22,23,24,25^ with self-reported information. To our knowledge, no observational studies have used total population data or considered different levels of severity of mental health issues when evaluating the association between parental leave and maternal mental health. Overcoming these limitations is crucial for a better understanding of the protective role of parental leave on mental health.

Sweden is globally recognized for its generous parental leave scheme, providing all residents, regardless of citizenship, with as many as 480 days of paid leave per child until the child reaches 12 years of age or completes grade 5 of compulsory education.^26^ In most other countries, including Australia, France, the United Kingdom, and the US, paid parental leave is not universally provided.^27^ However, remuneration in Sweden is contingent on income and employment status prior to childbirth. Specifically, individuals receive approximately 80% of their income for 195 days (390 days per couple) if they have been in continuous employment for at least 8 months and have met an income threshold prior to their estimated delivery date. Many individuals also receive an additional 10% supplement provided by their employers through collective agreements.^28^ In contrast, individuals who do not meet these work conditions receive a basic flat-rate benefit. The remaining paid parental leave days are compensated at a minimum rate, which is the same for all individuals. The objective of this study was to evaluate the association between levels of paid parental leave benefits and maternal mental health in the postpartum period among first-time mothers in Sweden, when preconception mental health, income, and employment status were considered.

## Methods

This cohort study was performed as part of the project on the unintended consequences of Swedish parental leave policy (ParLeHealth).^29^ The study was approved by the Swedish Ethical Review Authority, which waived the need for informed consent due to data being pseudonymized, and followed the Strengthening the Reporting of Observational Studies in Epidemiology (STROBE) reporting guideline.

### Study Design and Population

The study relied on data from several Swedish registers. We used the Total Population Register,^30^ the Multigenerational Register, and the Medical Birth Register to identify first-time singleton births. The Medical Birth Register encompasses approximately 98% of all births in Sweden.^31^ We further refined our population by excluding adopted children using the Multigenerational Register, since the age at adoption is unknown. Information on compensation level for parental leave benefits was obtained from the Swedish Social Insurance Agency.^26^ For data on health outcomes, we used the National Patient Register,^32^ which provides information on hospitalizations and specialized outpatient care, and the National Prescribed Drug Register.^33^ Sociodemographic information was extracted from the Longitudinal Integrated Database for Health Insurance and Labour Market Studies (LISA).^34^ Last, data on region of birth were sourced from the Total Population Register.

**Figure 1.**
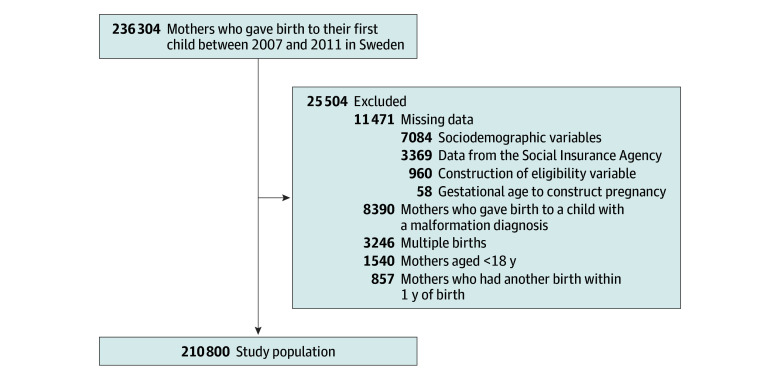
Study Selection

Between January 1, 2007, and December 31, 2011, a total of 236 304 mothers gave birth to their first child in Sweden. We excluded mothers who had multiple births (n = 3246), mothers who gave birth to their second child within 1 year of the first child (n = 857), those who delivered a child with a malformation diagnosis (n = 8390), and mothers younger than 18 years (n = 1540). After applying these exclusion criteria and excluding observations with missing data (n = 11 471), our final analytic population consisted of 210 800 mothers (Figure 1).

### Exposure

Parental leave benefit levels were determined by the compensation received when mothers first applied for parental leave. Mothers were categorized into 2 groups: those receiving higher-level benefits, qualifying for earnings-related benefits, and those receiving basic benefits, which are a low flat rate.

### Outcome

Maternal mental health during the postpartum period was assessed using 3 measures representing varying levels of severity and use of health care services (ie, mild, moderate, and severe): prescription of antidepressants or anxiolytics (mild), identified by Anatomical Therapeutic Chemical codes N06A and N05B, respectively; specialist outpatient care for mental disorders (moderate); and hospitalization for mental disorders (severe). Mental disorders for specialist outpatient care and hospital admission were identified by primary diagnosis using the *International Statistical Classification of Diseases, Tenth Revision*, codes F00 to F99. The measures were evaluated within the 1 year following childbirth. All outcomes were dichotomized.

### Covariables

Maternal age was grouped as 18 to 24, 25 to 29, 30 to 34, 35 to 39, and 40 years and older. Birth year of the child was obtained. Preconception mental health was defined as having been prescribed medication for depression or anxiety, receiving specialist outpatient care, or having been hospitalized for a mental disorder in the year before pregnancy. Maternal region of birth was categorized into (1) Sweden, (2) regions primarily composed of Organisation for Economic Cooperation and Development (OECD) member countries, and (3) non-OECD countries and regions listed as other.^35^ Maternal annual income was measured in the year before childbirth and categorized into quintiles. Maternal employment status, measured in November in the year before childbirth, was categorized into employed and not employed.

### Statistical Analysis

We used multivariable logistic regression to compare the odds of mental health outcomes between mothers receiving basic benefits compared with those receiving higher-level benefits. Results are presented as odd ratios (ORs) and 95% 95% CIs. Except for the unadjusted model, all models include birth year of the child to account for cohort variables and maternal age at childbirth (model 1). Model 2 additionally adjusted for preconception mental health, as prospective observational evidence shows that a large proportion of postpartum depression stems from preconception mental health.^3,4^ Model 3 added maternal income and employment status to determine the independent association with benefit levels.

We performed a decomposition analysis on the association of varying levels of parental leave benefits with the mother’s mental health using the Karlson-Holm-Breen method.^36^ This method estimates the contribution of a given variable to the association of interest, overcoming rescaling or attenuation bias that can occur with nonlinear models.^37,38^

To address confounding by income in the association between parental leave benefit levels and mental health outcomes, we applied inverse probability weighting regression adjustment with overlap weights as a robustness check.^39^ This method emphasizes mothers with the most overlap in observed characteristics (including income) between parental leave benefit levels.

We also performed a series of sensitivity analyses: (1) including family disposable income instead of maternal income to determine whether family income confounds the association between parental leave benefit levels and mental health; (2) restricting the study population to mothers without a history of mental disorders to remove an important confounder; and (3) restricting the study population to mothers aged 25 to 34 years to create a more homogeneous health population. Finally, we performed subgroup analyses by maternal region of birth to ascertain whether potential differences of the main estimate were stronger in one group compared with another.

Statistical analyses were conducted from March 8, 2024, to February 18, 2025, using Stata/SE, version 15.0 or 18.0 (StataCorp LLC).^40^ Two-sided *P* < .05 indicated statistical significance.

## Results

Among the 210 800 first-time mothers (mean [SD] age, 28.6 [5.0] years), 35 255 (16.72%) received basic benefits, while 175 545 (83.28%) qualified for higher-level benefits (Table). The proportion of missing data was low (5.4%). The mean age (SD) at childbirth was 25.1 (5.3) years for mothers receiving basic benefits and 29.3 (4.7) years for those receiving higher-level benefits. Most Swedish-born mothers (154 815 of 173 312 [89.33%]) received higher-level benefits, while 12 387 of 17 802 migrants from OECD (69.58%) and 8343 of 19 686 migrants from non-OECD (42.38%) regions received higher-level benefits. Among mothers receiving higher-level benefits, 10 790 (6.15%) were in the lowest income quintile compared with 28 774 (81.62%) receiving basic benefits. Among the total study population, 11 072 mothers (5.25%) were prescribed antidepressant or anxiolytic medication, 4927 (2.34%) received specialist outpatient care for mental disorders, and 608 (0.29%) were admitted to hospital for mental disorders in the 12 months following childbirth. History of mental health care prior to conception differed by level of parental leave benefit: 4279 (12.14%) among mothers receiving basic benefits vs 13 381 (7.62%) among mothers receiving higher-level benefits.

**Table.** Participant Characteristics^a^

Characteristic	Participant group			SMD^b^
	All (N = 210 800)	Higher level of parental leave benefits (n = 175 545)	Basic level of parental leave benefits (n = 35 255)	
Mother’s age at birth, mean (SD) [range], y	28.6 (5.0) [18-52]	29.3 (4.7) [18-51]	25.1 (5.3) [18-52]	0.84
Age range, y				
18-24	47 456 (22.51)	28 245 (16.09)	19 211 (54.49)	0.90
25-29	75 052 (35.60)	65 974 (37.58)	9078 (25.75)	
30-34	62 476 (29.64)	57 864 (32.96)	4612 (13.08)	
35-39	21 727 (10.31)	19 845 (11.30)	1882 (5.34)	
≥40	4089 (1.94)	3617 (2.06)	472 (1.34)	
Birth year of the child^c^				
2007	40 673 (19.29)	34 265 (84.25)	6408 (15.75)	0.06
2008	42 005 (19.93)	35 225 (83.86)	6780 (16.14)	
2009	42 393 (20.11)	35 164 (82.95)	7229 (17.05)	
2010	44 055 (20.90)	36 122 (81.99)	7933 (18.01)	
2011	41 674 (19.77)	34 769 (83.43)	6905 (16.57)	
Region of origin^c^				
Sweden	173 312 (82.22)	154 815 (89.33)	18 497 (10.67)	0.88
OECD country	17 802 (8.44)	12 387 (69.58)	5415 (30.42)	
Non-OECD country	19 686 (9.34)	8343 (42.38)	11 343 (57.62)	
Mother’s income quintile^d^				
First (bottom quintile)	39 564 (18.77)	10 790 (6.15)	28 774 (81.62)	2.80
Second	42 585 (20.20)	36 827 (20.98)	5758 (16.33)	
Third	42 895 (20.35)	42 532 (24.23)	363 (1.03)	
Fourth	42 863 (20.33)	42 645 (24.29)	218 (0.62)	
Fifth (top quintile)	42 893 (20.35)	42 751 (24.35)	142 (0.40)	
Family disposable income quintile				
First (bottom quintile)	40 579 (19.25)	21 124 (12.03)	19 455 (55.18)	1.04
Second	42 486 (20.15)	38 358 (21.85)	4128 (11.71)	
Third	42 493 (20.16)	39 259 (22.36)	3234 (9.17)	
Fourth	42 534 (20.18)	37 630 (21.44)	4904 (13.91)	
Fifth (top quintile)	42 708 (20.26)	39 174 (22.32)	3534 (10.02)	
Employment status^d^				
Employed	170 754 (81.00)	164 626 (93.78)	6128 (17.38)	2.40
Not employed	40 046 (19.00)	10 919 (6.22)	29 127 (82.62)	
Mental health				
No antidepressant or anxiolytic medication	199 728 (94.75)	166 893 (95.07)	32 835 (93.14)	0.08
Antidepressant or anxiolytic medication	11 072 (5.25)	8652 (4.93)	2420 (6.86)	
No specialist outpatient care	205 873 (97.66)	172 177 (98.08)	33 696 (95.58)	0.14
Specialist outpatient care	4927 (2.34)	3368 (1.92)	1559 (4.42)	
Hospitalization				
No	210 192 (99.71)	175 145 (99.77)	35 047 (99.41)	0.06
Yes	608 (0.29)	400 (0.23)	208 (0.59)	
Mental health treatment prior to conception				
None	193 140 (91.62)	162 164 (92.38)	30 976 (87.86)	0.15
Treatment	17 660 (8.38)	13 381 (7.62)	4279 (12.14)	

Abbreviations: OECD, Organisation for Economic Cooperation and Development; SMD, standardized mean difference.

^a^ Data are presented as No. (%) of participants unless indicated otherwise.

^b^ A small difference was defined as 0.2 to 0.5; a moderate difference, 0.5 to 0.8; and a large difference, larger than 0.8.

^c^ Percentages are calculated by row.

^d^ Measured in the previous year to childbirth.

**Figure 2.**
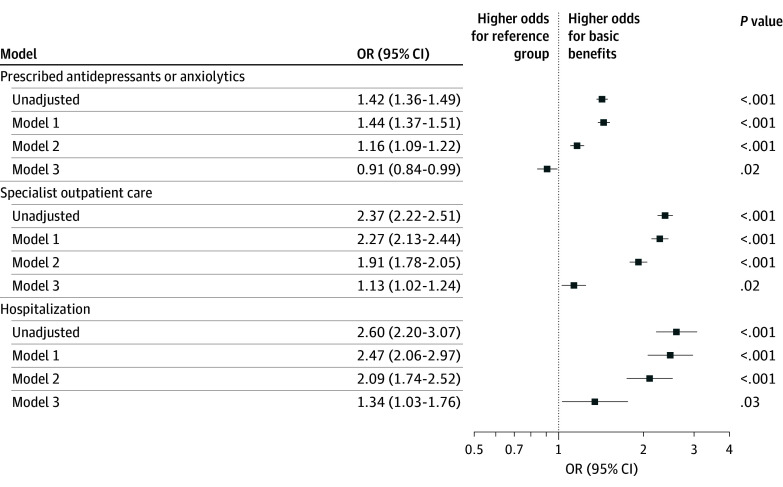
Association Between Paid Parental Leave Benefit Levels and Mental Health Outcomes in the 12 Months Following Childbirth Among First-Time Mothers Includes 210 800 registered births in Sweden. Model 1 was adjusted for maternal age and birth year of the child; model 2, for maternal age, birth year of the child, and preconception mental health; and model 3, for maternal age, birth year of the child, preconception mental health, and maternal income and employment status. OR indicates odds ratio.

Level of parental leave benefits was associated with maternal mental health in the year following childbirth (Figure 2 and eTable 1 in Supplement 1). Mothers receiving basic benefits had higher odds of being prescribed antidepressant or anxiolytic medication (OR, 1.44; 95% CI, 1.37-1.51), receiving specialist outpatient care for mental disorders (OR, 2.27; 95% CI, 2.13-2.44), and being hospitalized for mental disorders (OR, 2.47; 95% CI, 2.06-2.97) compared with mothers who received earnings-related benefits (model 1). Although preconception mental health was associated with all mental health outcomes, its inclusion only partly attenuated the odds and accounted for 46.1% of the association for prescribed antidepressants or anxiolytics (OR, 1.16; 95% CI, 1.09-1.22), 15.1% of the association for receiving specialist outpatient care (OR, 1.91; 95% CI, 1.78-2.05), and 10.7% of the association for hospitalization for mental disorders (OR, 2.09; 95% CI, 1.74-2.52) (model 2). After adjusting for income and employment status, the odds attenuated, but mothers receiving basic benefits still had higher odds of specialist outpatient care (OR, 1.13; 95% CI, 1.02-1.24) and for hospitalization (OR, 1.34; 95% CI, 1.03-1.76). Income and employment status explained 62.5% and 9.4% of the estimate for specialist outpatient care, respectively, and 34.4% and 21.3% of the estimate for hospitalization, respectively. In contrast, mothers receiving basic benefits had lower odds of being prescribed medication (OR, 0.91; 95% CI, 0.84-0.99) after accounting for income and employment status (model 3), with income contributing to 50.3% and employment status 7.1% of the association. The results of the decomposition analysis are displayed in eTable 2 in Supplement 1. Results from the robustness analyses showed similar patterns to those of the main analyses (eTables 3 and 4 in Supplement 1).

When we included family disposable income instead of maternal income and employment status, mothers receiving basic benefits had higher odds of being prescribed antidepressants or anxiolytics (OR, 1.06; 95% CI, 1.00-1.13), receiving specialist outpatient care (OR, 1.68; 95% CI, 1.56-1.82), and being hospitalized (OR, 1.88; 95% CI, 1.53-2.29) (model 3) (eTable 5 in Supplement 1). When we excluded mothers with a history of mental disorders or restricted the population to mothers aged 25 to 34 years (eTables 6 and 7 in Supplement 1), we observed similar patterns to the main analyses.

**Figure 3.**
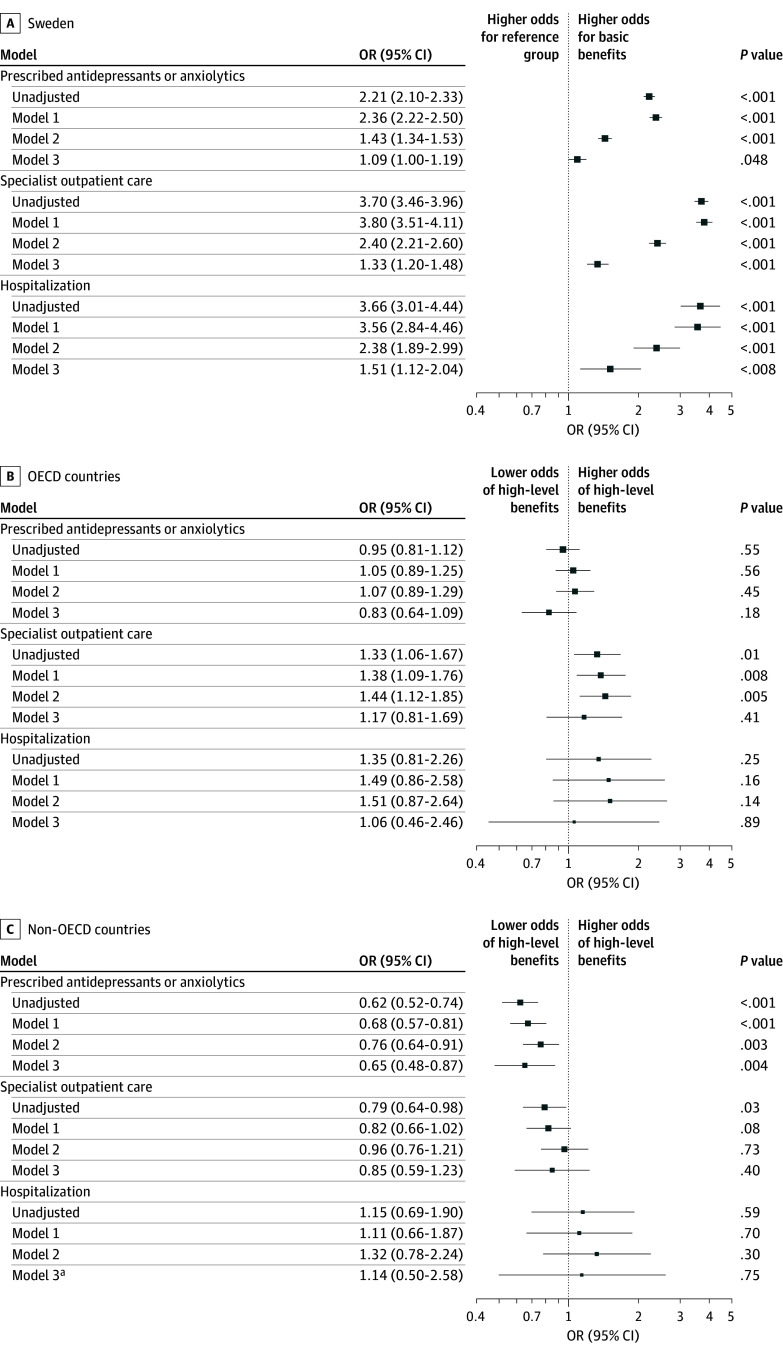
Association Between Paid Parental Leave Benefit Levels and Mental Health Outcomes in the 12 Months Following Childbirth Among First-Time Mothers Data are stratified by region of birth as Sweden (n = 173 312), Organisation for Economic Cooperation and Development (OECD) member countries (n = 17 802), and non-OECD countries (n = 19 686). Model 1 was adjusted for maternal age and birth year of the child; model 2, for maternal age, birth year of the child, and preconception mental health; and model 3, for maternal age, birth year of the child, preconception mental health, and maternal income and employment status. OR indicates odds ratio. ^a^The analytical sample was n = 18 417.

Swedish-born mothers receiving basic benefits had higher odds of prescribed antidepressants or anxiolytics (OR, 1.09; 95% CI, 1.00-1.19), receiving specialist outpatient care (OR, 1.33; 95% CI, 1.20-1.48), and being hospitalized (OR, 1.51; 95% CI, 1.12-2.04) compared with Swedish-born mothers receiving higher-level benefits (model 3 in Figure 3A). In contrast, no associations were found among foreign-born mothers for specialist outpatient care or hospitalizations (model 3 in Figure 3B and C). However, mothers from non-OECD regions receiving basic benefits had lower odds of being prescribed antidepressants or anxiolytics (OR, 0.65; 95% CI, 0.48-0.87) (model 3 in Figure 3C).

## Discussion

The level of paid parental leave benefits in Sweden was associated with maternal mental health in the year after childbirth. Mothers receiving basic benefits had higher odds of being prescribed antidepressants or anxiolytics, receiving specialist outpatient care, and being hospitalized compared with mothers receiving higher-level benefits. These associations remained after adjusting for preconception health. When maternal income and employment status were taken into consideration, the odds were reduced for specialist outpatient care and hospitalization, but remained significant; however, for prescriptions of antidepressants or anxiolytics, mothers receiving basic benefits had lower odds. Moreover, associations varied by maternal region of birth: Swedish-born mothers receiving basic benefits had higher odds of all mental health outcomes than those with higher-level benefits, while mothers from non-OECD regions receiving basic benefits had lower odds of being prescribed antidepressants or anxiolytics.

Our study contributes knowledge on several fronts. First, our results not only support prior research showing that generous parental leave benefits were associated with better postpartum mental health, but they further suggest that the more generous parental leave is, the greater benefits for mother’s mental health may be, as we observed differences in mental health across levels of paid parental leave that are already generous from an international perspective.

Second, our study shows that the association between parental leave benefits and maternal mental health persists after considering preconception mental health, a factor many observational studies have not controlled for. Our study shows that the association remains when adjusting for preconception mental health or when restricting the study population to mothers without a history of mental disorders. This finding is significant because it suggests that generous parental leave may be protective against the onset of severe mental health outcomes.

Third, our findings suggest that generous parental leave benefits may protect against moderate-to-severe mental disorders. The differences in mental health outcomes across paid parental leave benefit levels highlight the importance of adequate financial support during the transition to parenthood. We also recognize that the strong link between labor market conditions and paid parental leave levels makes it difficult to examine the association between paid parental leave benefits and mental health. However, the fact that parental leave benefit levels remained associated with mental health after these adjustments (albeit attenuated) suggests that parental leave benefits may also have an independent association with mental health. This interpretation is further supported by our robustness analysis. We acknowledge that our findings are preliminary and encourage future research to use alternative study designs to better understand the causal mechanisms. We hypothesize that the comparative disadvantage between women receiving basic vs higher levels of benefits may be partially explained by shorter uptake of parental leave, as lower levels of remuneration may necessitate an earlier return to the labor market. Further studies should examine leave uptake patterns to confirm this hypothesis. Furthermore, the connection between employment status and paid leave shows that efforts to protect postpartum mental health must consider the social determinants of women’s health by adopting a Health in All Policies approach.^41^ In this context, improving labor market conditions (eg, reducing precarious employment) and relaxing work requirements for higher-level parental leave benefits would benefit postpartum mental health.

It is important to note that in the Swedish context, women with a very low income prior to childbearing may experience an economic improvement with the basic parental benefit level relative to their income prior to parental leave. Given that this situation is more common among immigrants from the non-OECD region, one could hypothesize that this relative advantage could explain the lack of association between parental leave benefit levels and mental health among immigrants from this region. Furthermore, parental leave may offer immigrant mothers opportunities for societal integration. A recent study of newly arrived immigrant mothers to Sweden found that part-time parental leave (1-14 d/mo) may promote integration by providing essential support and connections, especially when spent on education or language school, aiding future employment.^42^

### Strengths and Limitations

Our study has several strengths. First, to the best of our knowledge, this is the first study to evaluate the association between levels of paid parental leave benefits and maternal mental health in the postpartum period among individuals who are entitled to paid leave, as previous studies have often combined the effects of paid and unpaid parental leave (ie, time off work) or a combination of unpaid leave and no leave at all.^2^ Second, we used data from total population national registers, ensuring representation within the Swedish population. Third, the proportion of missing data (5.4%) was low, so we were able to capture more than 94% of first-time mothers who had singleton births between 2007 and 2011 in Sweden, again ensuring representativeness. Fourth, our study was able to capture a broad range of use of mental health care services, ranging from milder issues (with which individuals may refrain from seeking help due to barriers) to severe issues requiring hospitalization (for which individuals ultimately require help regardless of barriers). Fifth, all health information was based on diagnoses or prescribed medication by physicians recorded in their respective registers, thereby avoiding recall bias. In addition, mothers routinely attend child health clinics to follow the child’s development. At these clinics, there are opportunities for mothers to be assessed for postpartum depression and referred to an appropriate health professional. Sixth, our study accounted for preconception mental health in the year prior to pregnancy using information from national registers. Finally, by directly accessing information on parental leave from the Swedish social insurance system, we were able to measure the utilization of parental leave rather than eligibility, as many previous studies have done.

This study also has some limitations. First, while we were unable to use a more detailed birthplace categorization, our categorization aligns with that of previous studies,^35,43^ thereby ensuring comparability. Second, because the LISA database reports annual rather than monthly income, we measured income in the year preceding childbirth. This serves as a suitable proxy, because income is protected 6 months before childbirth for eligibility for earnings-related parental leave benefits. Thus, an individual’s access to higher-level parental leave and their income will not be affected if they reduce their working hours, take extended sick leave, or become unemployed. Third, since LISA reports employment status only in November of each year, our measure is an approximation. Fourth, we were not able to account for the duration of leave to further investigate possible protective mechanisms.

## Conclusions

This cohort study shows that paid parental leave benefit levels are associated with maternal mental health in the postpartum period, with more generous benefits associated with better mental health, particularly moderate-to-severe mental disorders. However, since eligibility for higher-level parental leave benefits is contingent on labor market attachment, both improving women’s labor market attachment and relaxing stringent work requirements for generous parental leave could promote mental health among mothers.

## Notes

Supplement 1. **eTable 1.** Main Analyses: The Association Between Paid Parental Leave Benefit Levels and Postpartum Mental Health Outcomes Among First-Time Mothers Who Gave Birth Between 2007 and 2011 in Sweden (N = 210 800) **eTable 2.** Decomposition Analysis for the Association Between Paid Parental Leave Benefit Levels and Mental Health Risk in the 12 Months Following Childbirth Among First-Time Mothers Who Gave Birth Between 2007 and 2011 in Sweden Using the KHB Method (N = 210 800) **eTable 3.** Robustness Analysis: Results of Association Between Parental Leave Benefit Levels and Maternal Mental Health Using Inverse Probability Weighting Robust Adjustments With Overlap Weights (N = 210 800) **eTable 4.** Robustness Analysis: Postestimation Covariate Balance Summary **eTable 5.** The Association Between Parental Leave Benefit Levels and Mental Health Outcomes in the 12 Months Following Childbirth Among First-Time Mothers Who Gave Birth Between 2007 and 2011 in Sweden (N = 210 800) **eTable 6.** The Association Between Parental Leave Benefit Levels and Postpartum Mental Health Outcomes Among First-Time Mothers Without a History of Mental Health Care Who Gave Birth Between 2007 and 2011 in Sweden (n = 193 140) **eTable 7.** The Association Between Parental Leave Benefit Levels and Postpartum Mental Health Outcomes Among First-Time Mothers Aged 25 to 34 Years Who Gave Birth Between 2007 and 2011 in Sweden (n = 137 528)

Supplement 2.
Data Sharing Statement
