# Supplementary material for: Parental Leave Benefits and Maternal Postpartum Mental Health in Sweden
Source: JAMA Netw Open. 2025 Apr 30;8(4):e258062. doi: 10.1001/jamanetworkopen.2025.8062 (PMC12044515; doi:10.1001/jamanetworkopen.2025.8062)
Supplement: Supplement 1. — eTable 1. Main Analyses: The Association Between Paid Parental Leave Benefit Levels and Postpartum Mental Health Outcomes Among First-Time Mothers Who Gave Birth Between 2007 and 2011 in Sweden (N = 210 800) eTable 2. Decomposition Analysis for the Association Between Paid Parental Leave Benefit Levels and Mental Health Risk in the 12 Months Following Childbirth Among First-Time Mothers Who Gave Birth Between 2007 and 2011 in Sweden Using the KHB Method (N = 210 800) eTable 3. Robustness Analysis: Results of Association Between Parental Leave Benefit Levels and Maternal Mental Health Using Inverse Probability Weighting Robust Adjustments With Overlap Weights (N = 210 800) eTable 4. Robustness Analysis: Postestimation Covariate Balance Summary eTable 5. The Association Between Parental Leave Benefit Levels and Mental Health Outcomes in the 12 Months Following Childbirth Among First-Time Mothers Who Gave Birth Between 2007 and 2011 in Sweden (N = 210 800) eTable 6. The Association Between Parental Leave Benefit Levels and Postpartum Mental Health Outcomes Among First-Time Mothers Without a History of Mental Health Care Who Gave Birth Between 2007 and 2011 in Sweden (n = 193 140) eTable 7. The Association Between Parental Leave Benefit Levels and Postpartum Mental Health Outcomes Among First-Time Mothers Aged 25 to 34 Years Who Gave Birth Between 2007 and 2011 in Sweden (n = 137 528) [file jamanetwopen-e258062-s001.pdf]

Supplementary Online Content

Heshmati A, Honkaniemi H, Fritzell S, Juárez SP. Parental leave benefits and maternal postpartum mental health in Sweden. *JAMA Netw Open.* 2025;8(4):e258062. doi:10.1001/jamanetworkopen.2025.8062

eTable 1. Main Analyses: The Association Between Paid Parental Leave Benefit Levels and Postpartum Mental Health Outcomes Among First-Time Mothers Who Gave Birth Between 2007 and 2011 in Sweden (N = 210 800)

eTable 2. Decomposition Analysis for the Association Between Paid Parental Leave Benefit Levels and Mental Health Risk in the 12 Months Following Childbirth Among First-Time Mothers Who Gave Birth Between 2007 and 2011 in Sweden Using the KHB Method (N = 210 800)

eTable 3. Robustness Analysis: Results of Association Between Parental Leave Benefit Levels and Maternal Mental Health Using Inverse Probability Weighting Robust Adjustments With Overlap Weights (N = 210 800)

eTable 4. Robustness Analysis: Postestimation Covariate Balance Summary

eTable 5. The Association Between Parental Leave Benefit Levels and Mental Health Outcomes in the 12 Months Following Childbirth Among First-Time Mothers Who Gave Birth Between 2007 and 2011 in Sweden (N = 210 800)

eTable 6. The Association Between Parental Leave Benefit Levels and Postpartum Mental Health Outcomes Among First-Time Mothers Without a History of Mental Health Care Who Gave Birth Between 2007 and 2011 in Sweden (n = 193 140)

eTable 7. The Association Between Parental Leave Benefit Levels and Postpartum Mental Health Outcomes Among First-Time Mothers Aged 25 to 34 Years Who Gave Birth Between 2007 and 2011 in Sweden (n = 137 528)

This supplementary material has been provided by the authors to give readers additional information about their work.

© 2025 Heshmati A et al. *JAMA Network Open*.

eTable 1. Main Analyses: The Association Between Paid Parental Leave Benefit Levels and Postpartum Mental Health Outcomes Among First-Time Mothers Who Gave Birth Between 2007 and 2011 in Sweden (N = 210 800)

	Unadjusted OR (95%CI)	Model 1 OR (95%CI)	Model 2 OR (95%CI)	Model 3 OR (95%CI)
**Prescribed antidepressant or anxiolytic medication**				
Parental leave benefits				
Higher-level	1	1	1	1
Basic	1.42 (1.36-1.49) ***	1.44 (1.37-1.51) ***	1.16 (1.09-1.22) ***	0.91 (0.84-0.99) *
Mother's age				
18-24 years		1	1	1
25-29 years		0.91 (0.87-0.97) **	1.02 (0.98-1.08)	1.10 (1.04-1.17) **
30-34 years		1.04 (0.98-1.09)	1.13 (1.06-1.20) ***	1.34 (1.26-1.43) ***
35-39 years		1.25 (1.16-1.34) ***	1.26 (1.16-1.36) ***	1.51 (1.39-1.63) ***
40+ years		1.37 (1.21-1.56) ***	1.26 (1.09-1.45) **	1.48 (1.28-1.71) ***
Birth year				
2007		1	1	1
2008		1.07 (1.01-1.14) *	1.01 (0.95-1.09)	1.04 (0.97-1.12)
2009		1.16 (1.09-1.24) ***	1.11 (1.04-1.19) **	1.16 (1.08-1.24) ***
2010		1.22 (1.15-1.30) ***	1.15 (1.08-1.23) ***	1.21 (1.13-1.30) ***
2011		1.29 (1.21-1.37) ***	1.20 (1.12-1.28) ***	1.27 (1.18-1.36) ***
Preconception mental healthcare				
No			1	1
Yes			19.97 (19.14-20.83) ***	18.70 (17.92-19.52) ***
Mother's income				
First quintile (bottom)				1
Second				0.96 (0.88-1.05)
Third				0.77 (0.70-0.86) ***
Fourth				0.66 (0.59-0.73) ***
Fifth (top)				0.52 (0.46-0.58) ***
Employment status				
Employed				1
Not employed				1.03 (0.94-1.13)
**Specialist outpatient care for mental disorders**				
Parental leave benefits				
Higher-level	1	1	1	1
Basic	2.37 (2.22-2.51) ***	2.27 (2.13-2.44) ***	1.91 (1.78-2.05) ***	1.13 (1.02-1.24) *
Mother's age				
18-24 years		1	1	1
25-29 years		0.84 (0.78-0.91) ***	0.94 (0.86-1.01)	1.02 (0.94-1.10)
30-34 years		0.88 (0.81-0.95) **	0.94 (0.86-1.03)	1.13 (1.04-1.24) **
35-39 years		1.13 (1.02-1.25) *	1.12 (1.00-1.24) *	1.33 (1.20-1.49) ***
40+ years		1.24 (1.02-1.50) *	1.11 (0.91-1.36)	1.27 (1.04-1.55) *
Birth year				
2007		1	1	1
2008		1.07 (0.97-1.18)	1.01 (0.92-1.12)	1.05 (0.95-1.16)
2009		1.10 (1.01-1.21) *	1.05 (0.95-1.15)	1.11 (1.01-1.23) *
2010		1.18 (1.08-1.29) ***	1.10 (1.00-1.21) †	1.16 (1.06-1.28) **
2011		1.25 (1.14-1.37) ***	1.13 (1.03-1.24) *	1.21 (1.10-1.33) ***

© 2025 Heshmati A et al. *JAMA Network Open*.

Preconception mental healthcare				
No			1	1
Yes			15.78 (14.88-16.75) ***	14.33 (13.50-15.22) ***
Mother's income				
First quintile (bottom)				1
Second				0.75 (0.67-0.84) ***
Third				0.54 (0.47-0.62) ***
Fourth				0.41 (0.35-0.47) ***
Fifth (top)				0.40 (0.34-0.46) ***
Employment status				
Employed				1
Not employed				1.11 (0.98-1.25)
**Hospitalisation for mental disorders**				
Parental leave benefits				
Higher-level	1	1	1	1
Basic	2.60 (2.20-3.07) ***	2.47 (2.06-2.97) ***	2.09 (1.74-2.52) ***	1.34 (1.03-1.76) *
Mother's age				
18-24 years		1	1	1
25-29 years		0.75 (0.61-0.94) *	0.82 (0.66-1.02)	0.86 (0.69-1.07)
30-34 years		0.85 (0.68-1.08)	0.91 (0.72-1.14)	1.05 (0.83-1.33)
35-39 years		1.15 (0.86-1.52)	1.14 (0.86-1.52)	1.33 (1.00-1.78)
40+ years		1.55 (0.96-2.50)	1.43 (0.88-2.31)	1.62 (1.00-2.63) †
Birth year				
2007		1	1	1
2008		1.04 (0.80-1.34)	0.99 (0.77-1.28)	1.02 (0.79-1.32)
2009		0.94 (0.73-1.22)	0.90 (0.70-1.17)	0.95 (0.73-1.23)
2010		1.02 (0.79-1.31)	0.96 (0.75-1.23)	1.00 (0.78-1.29)
2011		0.99 (0.76-1.27)	0.91 (0.70-1.17)	0.96 (0.74-1.24)
Preconception mental healthcare				
No			1	1
Yes			8.52 (7.25-10.02) ***	7.86 (6.67-9.26) ***
Mother's income				
First quintile (bottom)				1
Second				0.85 (0.61-1.18)
Third				0.78 (0.53-1.14)
Fourth				0.62 (0.41-0.93) *
Fifth (top)				0.46 (0.30-0.71) **
Employment status				
Employed				1
Not employed				1.27 (0.92-1.77)

OR: odds ratio; 95%CI: 95% confidence intervals, †p=0.05; *p<0·05; **p<0·01; ***p<0·001
Mother's income measured in the previous year to childbirth.

© 2025 Heshmati A et al. *JAMA Network Open*.

eTable 2. Decomposition Analysis for the Association Between Paid Parental Leave Benefit Levels and Mental Health Risk in the 12 Months Following Childbirth Among First-Time Mothers Who Gave Birth Between 2007 and 2011 in Sweden Using the KHB Method (N = 210 800)

	Effect	Model 2<br>OR (95% CI)	Model 3<br>OR (95% CI)
**Antidepressant or anxiolytic medication**			
Parental leave benefits			
Higher-level		1	1
Basic		1.16 (1.09-1.22) ***	0.91 (0.84-0.99) *
	Effect reduction by preconception mental health	46.1%	40.3%
	Effect reduction by income		50.3%
	Effect reduction by employment status		7.1%
**Specialist outpatient care for mental disorders**			
Parental leave benefits			
Higher-level		1	1
Basic		1.91 (1.78-2.05) ***	1.13 (1.02-1.24) *
	Effect reduction by preconception mental health	15.1%	13.5%
	Effect reduction by income		62.5%
	Effect reduction by employment status		9.4%
**Hospitalisation for mental disorders**			
Parental leave benefits			
Higher-level		1	1
Basic		2.09 (1.74-2.52) ***	1.34 (1.03-1.76) *
	Effect reduction by preconception mental health	10.7%	10.0%
	Effect reduction by income		34.4%
	Effect reduction by employment status		21.3%

OR: odds ratio; 95%CI: 95% confidence intervals, *p<0·05; ***p<0·001
Model 2: Adjusted for mother's age, birth year of the child, preconception mental health
Model 3: Adjusted for mother's age, birth year of the child, preconception mental health, mother's income and employment status

© 2025 Heshmati A et al. *JAMA Network Open*.

eTable 3. Robustness Analysis: Results of Association Between Parental Leave Benefit Levels and Maternal Mental Health Using Inverse Probability Weighting Robust Adjustments With Overlap Weights (N = 210 800)

	**ATE coefficient (95% CI)**	**PO mean (95%CI)**
**By parental leave benefit level**<br>(Reference: higher-level benefits)		
Prescribed antidepressants or anxiolytics	-0.0059618 (-0.0104676, -0.0014561) *	0.0740529 (0.070907, 0.0771988) ***
Specialist outpatient care	0.0038379 (0.0004187, 0.0072571) *	0.0364263 (0.034047, 0.0388057) ***
Hospitalisation	0.0014023 (0.0001498, 0.0026548) *	0.0038393 (0.0030405, 0.004638) ***

*p<0·05; **p<0·01; ***p<0·001

ATE: average treatment effect for those receiving basic benefits, 95% CI: 95% confidence interval; PO mean: estimated average outcome for those receiving earnings-related benefits. Inverse probability weighting score of parental leave benefit levels) calculated based on mother's age, child's year of birth, preconception mental health, income in quintiles and employment status.

© 2025 Heshmati A et al. *JAMA Network Open*.

eTable 4. Robustness Analysis: Postestimation Covariate Balance Summary

**Summary**	**Raw**	**Weighted**		
Number of observations	210,800	210,800.0		
Treated observations (*Basic benefits*)	35,255	115,369.1		
Control observations (*Higher-level benefits*)	175,545	95,430.9		
	**Standardised differences**		**Variance ratio**	
	**Raw**	**Weighted**	**Raw**	**Weighted**
Mother's age				
25-29 years	-0.2564557	0.1240924	0.8150551	1.059676
30-34 years	-0.4859927	0.036978	0.5145779	1.031825
35-39 years	-0.2172866	-0.0864808	0.5039883	0.7801859
40+ years	-0.0558498	-0.0387227	0.6545748	0.7461027
Birth year				
2008	-0.0210098	0.0143555	0.9684299	1.02145
2009	0.0117803	-0.0324148	1.017602	0.9510816
2010	0.0468315	-0.0468406	1.067061	0.9319484
2011	-0.0055429	-0.0722426	0.9916108	0.8887569
Prior history of mental health	0.1517355	-0.0044281	1.514502	0.9865731
Income				
Quintile 2	-0.1194821	-0.0259722	0.8243197	0.9614817
Quintile 3	-0.7453044	0.141842	0.0555096	1.196403
Quintile 4	-0.7679808	-0.0096212	0.0334146	0.9858222
Quintile 5	-0.780687	-0.0381812	0.0217759	0.9433503
Not employed	2.404271	-0.0880792	2.461946	0.8577525

© 2025 Heshmati A et al. *JAMA Network Open*.

eTable 5. The Association Between Parental Leave Benefit Levels and Mental Health Outcomes in the 12 Months Following Childbirth Among First-Time Mothers Who Gave Birth Between 2007 and 2011 in Sweden (N = 210 800)

	**Unadjusted OR (95%CI)**	**Model 1 OR (95%CI)**	**Model 2 OR (95%CI)**	**Model 3 OR (95%CI)**
**Prescribed antidepressant or anxiolytic medication**				
Parental leave benefits				
Higher-level	1	1	1	1
Basic	1.42 (1.36-1.49) ***	1.44 (1.37-1.51) ***	1.16 (1.09-1.22) ***	1.06 (1.00-1.13) *
**Specialist outpatient care for mental disorders**				
Parental leave benefits				
Higher-level	1	1	1	1
Basic	2.37 (2.22-2.51) ***	2.27 (2.13-2.44) ***	1.91 (1.78-2.05) ***	1.68 (1.56-1.82) ***
**Hospitalisation for mental disorders**				
Parental leave benefits				
Higher-level	1	1	1	1
Basic	2.60 (2.20-3.07) ***	2.69 (2.24-3.22) ***	2.23 (1.86-2.68) ***	1.88 (1.53-2.29) ***

OR: odds ratio; 95%CI: 95% confidence intervals, *p<0·05; **p<0·01; ***p<0·001
Model 1: Adjusted for mother's age and birth year of the child
Model 2: Adjusted for mother's age, birth year of the child, and preconception mental health
Model 3: Adjusted for mother's age, birth year of the child, preconception mental health, and family disposable income
Family disposable income measured in the previous year to childbirth

© 2025 Heshmati A et al. *JAMA Network Open*.

eTable 6. The Association Between Parental Leave Benefit Levels and Postpartum Mental Health Outcomes Among First-Time Mothers Without a History of Mental Health Care Who Gave Birth Between 2007 and 2011 in Sweden (n = 193 140)

	Unadjusted OR (95%CI)	Model 1 OR (95%CI)	Model 2 OR (95%CI)	Model 3 OR (95%CI)
**Prescribed antidepressant or anxiolytic medication**				
Parental leave benefits				
Earnings-related	1	1	1	1
Basic	1.37 (1.28-1.47) ***	1.28 (1.19-1.38) ***	0.97 (0.87-1.08)	1.13 (1.05-1.23) **
Mother's age				
18-24 years		1	1	1
25-29 years		0.79 (0.73-0.85) ***	0.89 (0.83-0.97) **	0.84 (0.78-0.91) ***
30-34 years		0.84 (0.78-0.92) ***	1.09 (1.00-1.18)	0.95 (0.87-1.03)
35-39 years		0.95 (0.86-1.06)	1.24 (1.11-1.38) ***	1.08 (0.97-1.20)
40+ years		0.93 (0.76-1.15)	1.18 (0.96-1.46) *	1.05 (0.85-1.30)
Birth year				
2007		1	1	1
2008		0.96 (0.87-1.05)	0.99 (0.90-1.08)	0.98 (0.90-1.08)
2009		1.04 (0.95-1.14)	1.10 (1.00-1.20) *	1.08 (0.99-1.19)
2010		1.09 (1.00-1.19)	1.15 (1.05-1.26) **	1.14 (1.04-1.25) **
2011		1.11 (1.02-1.22) *	1.19 (1.08-1.30) ***	1.17 (1.07-1.28) **
Mother's income				
First quintile (bottom)			1	
Second			1.03 (0.91-1.17)	
Third			0.75 (0.65-0.87) ***	
Fourth			0.60 (0.52-0.70) ***	
Fifth (top)			0.47 (0.40-0.54) ***	
Employment status				
Employed			1	
Not employed			1.03 (0.90-1.17)	
Family disposable income				
First (bottom)				1
Second				0.83 (0.76-0.91) ***
Third				0.68 (0.62-0.75) ***
Fourth				0.65 (0.59-0.72) ***
Fifth (top)				0.65 (0.59-0.72) ***
**Specialist outpatient care for mental disorders**				
Parental leave benefits				
Earnings-related	1	1	1	1
Basic	1.87 (1.70-2.07) ***	1.72 (1.54-1.91) ***	1.16 (1.00-1.36)	1.51 (1.341.69) ***
Mother's age				
18-24 years		1	1	1
25-29 years		0.78 (0.70-0.88) ***	0.87 (0.78-0.98)	0.83 (0.74-0.93) **
30-34 years		0.75 (0.67-0.85) ***	0.94 (0.83-1.07)	0.83 (0.73-0.94) **
35-39 years		0.97 (0.83-1.14)	1.21 (1.03-1.42) *	1.07 (0.91-1.25)
40+ years		0.87 (0.63-1.20)	1.05 (0.76-1.46)	0.95 (0.69-1.32)

© 2025 Heshmati A et al. *JAMA Network Open*.

Birth year				
2007		1	1	1
2008		0.96 (0.84-1.11)	0.99 (0.86-1.14)	0.99 (0.86-1.13)
2009		1.02 (0.89-1.16)	1.07 (0.93-1.23)	1.05 (0.92-1.21)
2010		1.07 (0.93-1.22)	1.12 (0.98-1.28)	1.11 (0.97-1.27)
2011		1.09 (0.95-1.25)	1.15 (1.00-1.32) *	1.14 (0.99-1.30)
Mother's income				
First quintile (bottom)			1	
Second			0.96 (0.80-1.16)	
Third			0.69 (0.55-0.85) **	
Fourth			0.53 (0.43-0.67) ***	
Fifth (top)			0.49 (0.40-0.62) ***	
Employment status				
Employed			1	
Not employed			1.11 (0.92-1.33)	
Family disposable income				
First (bottom)				1
Second				0.79 (0.69-0.91) **
Third				0.63 (0.55-0.73) ***
Fourth				0.69 (0.60-0.79) ***
Fifth (top)				0.69 (0.60-0.80) ***
**Hospitalisation for mental disorders**				
Parental leave benefits				
Earnings-related	1	1	1	1
Basic	2.27 ((1.80-2.87) ***	2.07 (1.60-2.68) ***	1.44 (0.98-2.13)	1.83 (1.39-2.42) ***
Mother's age				
18-24 years		1	1	1
25-29 years		0.80 (0.60-1.07)	0.84 (0.63-1.12)	0.84 (0.63-1.12)
30-34 years		0.72 (0.52-0.99) *	0.83 (0.60-1.15)	0.79 (0.57-1.08)
35-39 years		0.84 (0.60-1.34)	1.04 (0.69-1.58)	0.97 (0.64-1.47)
40+ years		1.30 (0.65-2.60)	1.49 (0.75-2.99)	1.41 (0.70-2.81)
Birth year				
2007		1	1	1
2008		1.16 (0.84-1.61)	1.19 (0.86-1.64)	1.19 (0.86-1.65)
2009		0.90 (0.64-1.27)	0.93 (0.66-1.31)	0.93 (0.66-1.31)
2010		0.87 (0.62-1.23)	0.90 (0.64-1.27)	0.91 (0.64-1.28)
2011		0.84 (0.59-1.19)	0.87 (0.61-1.24)	0.87 (0.61-1.25)
Mother's income				
First quintile (bottom)			1	
Second			1.03 (0.64-1.67)	
Third			1.00 (0.57-1.77)	
Fourth			0.85 (0.60-1.53)	
Fifth (top)			0.64 (0.35-1.18)	
Employment status				
Employed			1	

© 2025 Heshmati A et al. *JAMA Network Open*.

Not employed	1.44 (0.89-2.34)	
Family disposable income		
First (bottom)		1
Second		0.83 (0.59-1.15)
Third		0.66 (0.46-0.95) *
Fourth		0.79 (0.56-1.11)
Fifth (top)		0.62 (0.42-0.90) *

OR: odds ratio; 95%CI: 95% confidence intervals, *p<0·05; **p<0·01; ***p<0·001

eTable 7. The Association Between Parental Leave Benefit Levels and Postpartum Mental Health Outcomes Among First-Time Mothers Aged 25 to 34 Years Who Gave Birth Between 2007 and 2011 in Sweden (n = 137 528)

	**Unadjusted OR (95%CI)**	**Model 1 OR (95%CI)**	**Model 2 OR (95%CI)**	**Model 3 OR (95%CI)**
**Prescribed antidepressant or anxiolytic medication**				
Parental leave benefits				
Higher-level	1	1	1	1
Basic	1.45 (1.35-1.56) ***	1.48 (1.37-1.59) ***	1.19 (1.10-1.29) ***	0.84 (0.75-0.94) **
**Specialist outpatient care for mental disorders**				
Parental leave benefits				
Higher-level	1	1	1	1
Basic	2.41 (2.19-2.64) ***	2.43 (2.21-2.67) ***	2.04 (1.85-2.26) ***	1.11 0.97-1.27)
**Hospitalisation for mental disorders**				
Parental leave benefits				
Higher-level	1	1	1	1
Basic	2.29 (1.74-3.00) ***	2.33 (1.77-3.07) ***	1.97 (1.50-2.60) ***	1.12 (0.76-1.65)

OR: odds ratio; 95%CI: 95% confidence intervals, *p<0·05; **p<0·01; ***p<0·001
Model 1: Adjusted for mother's age (continuous) and birth year of the child
Model 2: Adjusted for mother's age (continuous), birth year of the child, and preconception mental health
Model 3: Adjusted for mother's age (continuous), birth year of the child, preconception mental health, mother's income and employment status

© 2025 Heshmati A et al. *JAMA Network Open*.
