# Supplementary material for: Parental Leave Benefits and Maternal Postpartum Mental Health in Sweden
Source: JAMA Netw Open. 2025 Apr 30;8(4):e258062. doi: 10.1001/jamanetworkopen.2025.8062 (PMC12044515; doi:10.1001/jamanetworkopen.2025.8062)
Supplement: Supplement 2. — Data Sharing Statement [file jamanetwopen-e258062-s002.pdf]

Data Sharing Statement

Heshmati. Parental Leave Benefits and Maternal Postpartum Mental Health in Sweden. *JAMA Netw Open*. Published April 30, 2025. doi:10.1001/jamanetworkopen.2025.8062

Data
Data available: No

Additional Information
Explanation for why data not available: The data are available from Statistics Sweden under license for the current study, and are not publicly available.
